# Laser Therapy for Incision Healing in 9 Dogs

**DOI:** 10.3389/fvets.2018.00349

**Published:** 2019-01-29

**Authors:** Jennifer L. Wardlaw, Krista M. Gazzola, Amanda Wagoner, Erin Brinkman, Joey Burt, Ryan Butler, Julie M. Gunter, Lucy H. Senter

**Affiliations:** ^1^Department of Clinical Sciences, Animal Health Center, College of Veterinary Medicine, Mississippi State University, Starkville, MS, United States; ^2^Gateway Veterinary Surgery, St. Louis, MO, United States; ^3^Office of Research and Economic Development, Department of Clinical Sciences, College of Veterinary Medicine, Mississippi State University, Starkville, MS, United States

**Keywords:** laser, wound healing, canine, scar scale, IVDD, incision, photobiomodulation

## Abstract

Laser therapy is becoming common place in veterinary medicine with little evidence proving efficacy or dosages. This study evaluated surgical wound healing in canines. Twelve Dachshunds underwent thoraco-lumbar hemilaminectomies for intervertebral disc disease (IVDD). Digital photographs were taken of their incisions within 24 h of surgery and 1, 3, 5, 7, and 21 days postoperatively. The first three dogs were used to create a standardized scar scale to score the other dogs' incision healing. The remaining 9 dogs were randomly assigned to either receive 8 J/cm^2^ laser therapy once a day for 7 days or the non-laser treated control group. Incision healing was scored based on the scar scale from 0 to 5, with zero being a fresh incision and five being completely healed with scar contraction and hair growth. All scar scores significantly improved with increasing time from surgery (<0.001). Good agreement was achieved for inter-rater reliability (*p* = 0.9). Laser therapy increased the scar scale score, showed improved cosmetic healing, by day seven and continued to be significantly increased on day 21 compared to control dogs (*p* < 0.001). Daily application of laser therapy at 8J/cm2 hastened wound healing in Dachshunds that received thoracolumbar hemilaminectomies for IVDD. It also improved the cosmetic appearance.

## Introduction

Laser therapy is a novel rehabilitation technique being used in veterinary medicine for both rehabilitation and therapeutic purposes. Photobiomodulation (PBM) induced by laser therapy is the application of electromagnetic radiation within the near infrared spectrum and is aimed at stimulating healing or analgesia within the target tissue. Currently laser therapy is being advocated for a variety of conditions some of which include musculoskeletal pain, osteoarthritis, joint pain, and inflammation, neuropathic pain, otitis, dermatitis, chronic, or non-healing wounds and decubital ulcers (1–5).

There are three phases of wound healing; the inflammatory, proliferative and remodeling phases. The inflammatory phase is initiated at the time of injury and begins with hemostasis and formation of the platelet plug. Platelets release platelet-derived growth factor which attract neutrophils and more importantly macrophages. Macrophages attract fibroblasts and therefore commence the proliferative phase. Fibroblasts differentiate into myofibroblasts and cause tissue contraction. Tensile strength is increased by collagen reorganization and the eventual outcome is a wound that reaches 80% of the strength of uninjured tissue (6, 7). It has been shown in experimental studies that laser therapy reduces pain, positively influences inflammatory, proliferative, and maturation phases of wound healing and increases wound tensile strength (6, 8–10). However, most of these studies have been in laboratory animals and do not account for the difference in wound healing between species.

Despite the numerous accounts of the potential positive effects of laser therapy in various applications for both human and veterinary medicine, exact protocols for various conditions, and tissue healing do not exist. Recent studies in veterinary medicine show the potential benefit of wound healing using PBM induced by laser therapy, including accelerated wound healing in equine distal limb wounds using a wavelength of 635 nm and an energy output of 17 mW per diode for a power density of 5.1 J/cm2 (11). Another recent canine study has shown that surgery in combination with PBM decreases the time to ambulation in dogs with T3-L3 myelopathy secondary to intervertebral disk herniation using a wavelength of 810 nm and an energy output of 200 mW for a power density of 2–8 J/cm2 (4). Looking at other laser therapy reports in the literature wound healing protocols vary from 1 to 40 J/cm^2^, consequently necessitating the continued need for controlled research studies in order to evaluate the efficacy of proposed protocols using specified power densities on specific target tissues for defined clinical indications (8, 12–17).

This study attempts to objectively measure the ability of PBM induced by laser therapy to accelerate the healing time of surgically created wounds by using a previously described scar scale that corresponds with histopathology (18). This scar scale using photography has shown in numerous species that scar cosmetics is a consistent and sensitive indicator of histologic healing and is independent of the reviewer (3, 18–21). By using digital photographs, blinded to the reviewers, the present study evaluated healing while avoiding tissue sample collection. The goal of the study was to objectively evaluate the use of laser therapy as a treatment modality in canine intervertebral disc disease (IVDD) patients for surgical incisional healing.

## Materials and Methods

All procedures were approved by the Mississippi State University Institutional Animal Care and Use Committee and all treatment and control animal participants had documented informed client consent before enrollment in the study. The use of veterinarians to score the incision healing was approved by the Mississippi State University Institutional Review Board for the Protection of Human Subjects in Research.

Dachshund patients who present to College of Veterinary Medicine, Mississippi State University for a hemilaminectomy in the thoraco-lumbar region were included in this study. Dachshunds with a dapple colored coat, known systemic health issues, incisions closed with staples, or owners that did not consent were not included in the study. Dogs that had received previous back surgeries were also not included in the study population. Dogs that met the inclusion criteria had data collected with regards to signalment, weight, body condition score, coat color, location of disc herniation, surgical incision length, type of suture used, fat pad graft or gel foam usage, steroid administration, bladder medications, neurologic status at presentation, and at the time of discharge.

A 10 mega-pixel camera^1^ was used to take digital photographs. Photographs were taken at an equal distance (15 cm), angle, setting and lighting on days 0, 1, 3, 5, and 7 and again at day 21 of all dogs, with day 0 being the day of surgery. All photographs were taken with the standardized distance from the incision, on the same exam table, with the same camera settings and 90° from the dogs' back, by one of two authors (KG or AW).

**Figure 1:**
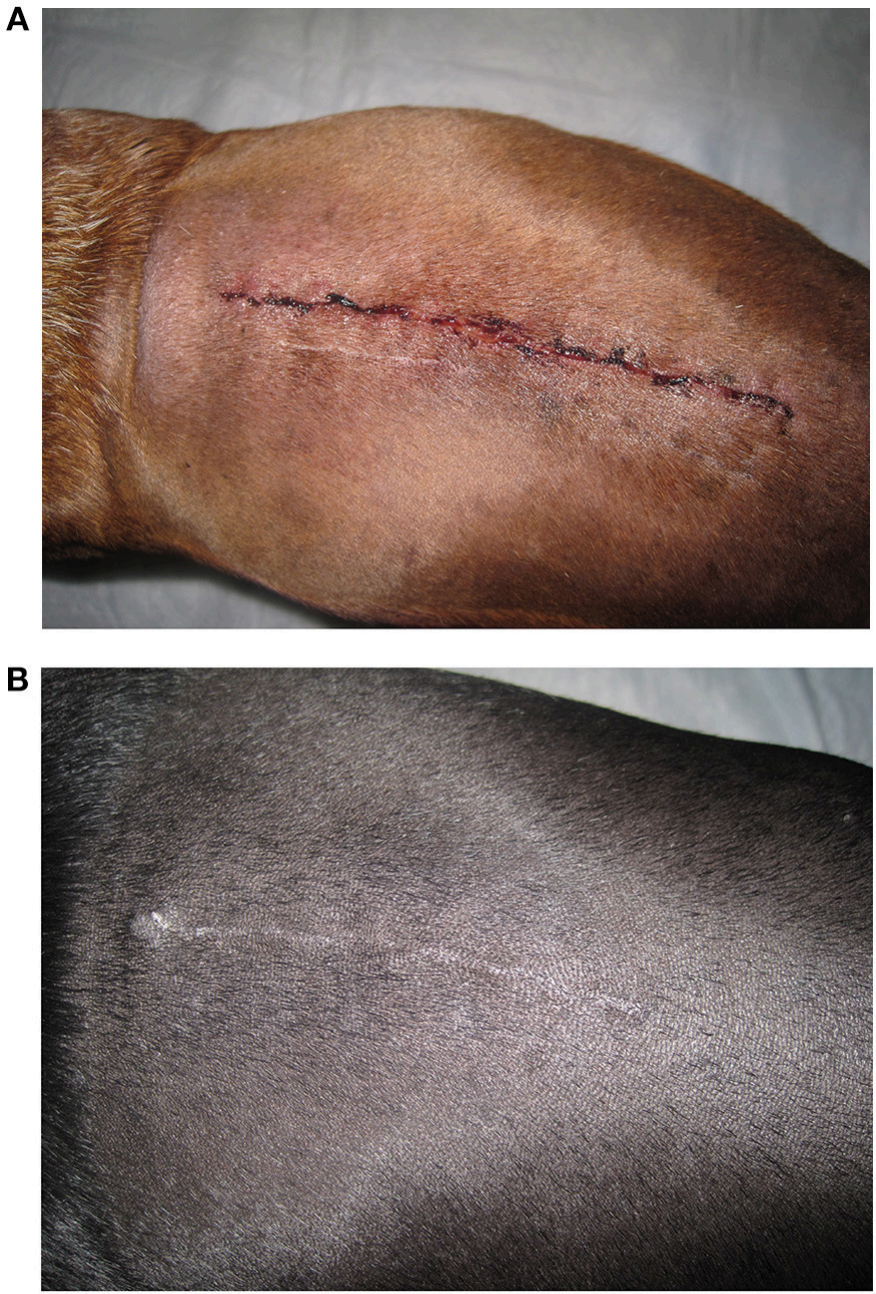
Examples of the scar scale images used for reviewer ranking of surgical incisions for both treatment groups. **(A)** This image was defined as a scar scale score of zero. **(B)** This image was defined as a scar scale score of five.

A clinical scar scale using digital photography was created as previously described (18). The first three dogs presented that met our inclusion criteria had digital photographs taken at days 0, 1, 3, 5, 7, and 21 using the standardized variables listed. These three dogs were used to create the scar scale from 0 to 5 with 0 being a fresh surgical incision (day 0 picture), a score of 1 with a fresh incision but no hemorrhage present (day 1 picture), a score of 2 had incision with some scabbing, swelling or bruising (day 2 picture), a score of 3 had visible healing with ongoing skin remodeling but resolving bruising or inflammation (day 5 picture), a score of 4 had healing progressing but a visible scar present (day 7 picture), and a score of five had a completely healed surgical incision with epithelialization, contraction and hair regrowth (day 21 picture) (Figures 1A,B).

The first three dogs enrolled in the study were selected and photographed to assure appropriate and expected healing without complication. After the initial three dogs were used to create the visually representative scar scale, the next qualifying dog was assigned to one of two treatment groups (laser or non-laser) using a coin flip. Subsequent dogs were then assigned every other to maintain similar treatment group sizes. Laser therapy dogs received 8 J/cm^2^ daily for seven days, beginning on day 1 using a class 3B veterinary laser^2^. The laser was wiped clean before and after each patient with the Novus polish system cloth provided by the laser manufacturer. The incision and one additional probe head spot size (7.55 cm^2^) around the entire incision (cranial, bilaterally the length of the incision, and caudal) were treated, except for over the laminectomy site. Total patient irradiation times varied due to varying lengths of incisions. The probe^3^ was applied with contact, but no pressure, perpendicular to the skin at all times used. The manufacturer pre-programmed muscle/increase local circulation for acute, low pulse setting was used. This setting has a pulsatile setting at 8 Hz with a 90% on, 10% off emissions at 850 nm for laser diodes and 670 nm for LED and defaults at 4 J/cm^2^. All treatment spots received 4 J/cm^2^ in this described spot pattern, twice during each treatment totally 8 J/cm^2^ treatment dosage. Digital photographs were taken at days 0, 1, 3, 5, 7, and 21. The non-laser group did not receive laser treatment, and had pictures taken at days 0, 1, 3, 5, 7, and 21. Upon completion of the study all pictures were randomly assigned a number, 1 through 125, using a computer generated^4^, unsorted list. The scar scale dog photographs were arranged on white cork board and labeled as 0, 1, 2, 3, 4, or 5 to define the scar scale. The board represented a score of 0 (day 0), 1 (day 1), 2 (day 3), 3 (day 5), 4 (day 7), and spaced linearly until the day 21 photographs which represented a scar scale score of 5. The treatment group photos were arranged in ascending numerical order according to their randomly assigned image number.

Veterinary volunteers, not involved in the surgery, patient care, laser therapy, or photograph acquisition were recruited to score the images (JW, EB, JB, RB, JG, LS). All scoring sessions were performed privately, in the same room and during daylight hours. All volunteers were instructed to score the images using whole integers from 0 to 5 based on cosmetic healing by considering; incision oozing, bruising, crusting, inflammation, edema, granulation, epithelialization, contraction, and hair regrowth on the incision site. Each volunteer scored all the photographs once and did so in one sitting. Once all six evaluators had ranked the images, the author (JW) was then given the treatment group assignments from the co-investigators (KG, AW) to send for statistical analysis.

The tests for significance of sources of variation and inter-rater reliability were determined using covariant analysis performed with Statistical Analysis System's GLIMMIX program^5^. The clinical importance of statistically significant differences in treatments was assessed using confidence intervals (22). A *P*-value of < 0.05 was considered to be significant. Free marginal Kappa values were used to assess inter-rater agreement.

## Results

This study was performed entirely at the Animal Health Center, College of Veterinary Medicine, Mississippi State University from September 2010 to May 2012. Twelve dogs met ouri inclusion criteria during this timeframe and were designated as three scar scale example dogs, five non-laser dogs, and four laser therapy dogs. Signalment, body condition score, incision length, lesion location, use of fat graft and/or gel foam, suture material used, steroid administration, and neurologic status at the time of surgery did not differ between laser therapy and non-laser dogs. All the laser therapy dogs were brown, whereas the non-laser group contained three black and two brown dogs. All dogs enrolled in the study appeared calm and comfortable for photography acquisition and laser therapy; therefore, were able to complete the study. All the dogs remained static in their neurologic status at the time of discharge or improved, none declined. Most of the dogs received steroids either at the time of surgery or before referral (67%). Some of the non-laser dogs (*n* = 4) and laser dogs (*n* = 2) received steroids. Steroid type, dosage, time of administration and frequency varied within the study population.

**Figure 2:**
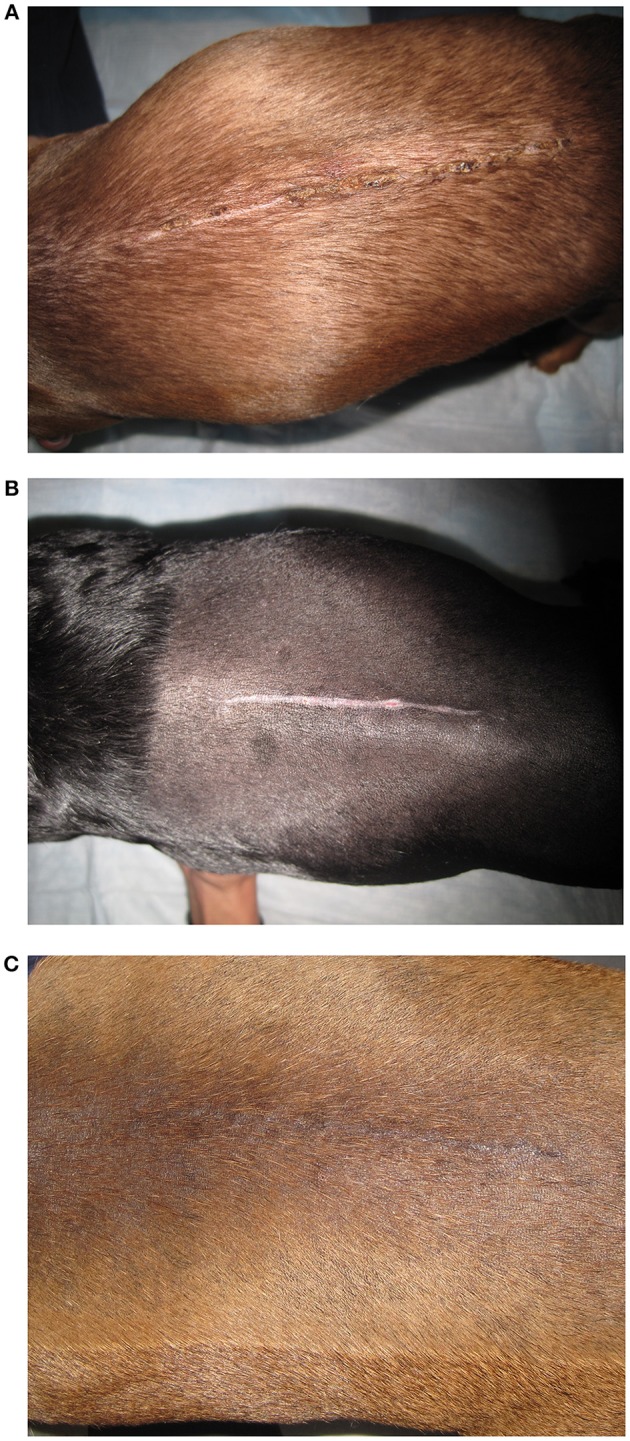
Representative images of the two treatments groups at day 21. **(A)** A non-laser patient illustrating the continued presence of a scab over some of the incision's epithelium. **(B)** A non-laser patient with a wide scar area and one remaining pink area of granulation toward the right side of the incision. **(C)** A laser therapy patient showing a completely healed incision with contraction and hair regrowth around and on the incision.

**Figure 3:**
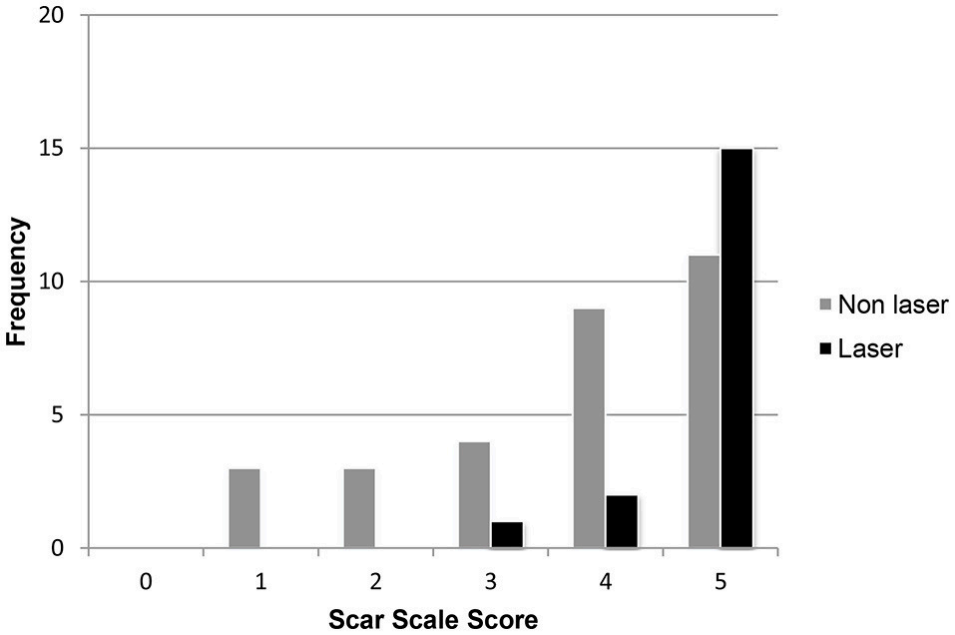
Frequency histogram showing individual scar scale scores (*n* = 48) on day 21 for the low intensity laser therapy (*n* = 3) and non-laser treatments (*n* = 5) groups from the six veterinary reviewers.

The first three dogs healed without incident, appeared uniformly cosmetic and were selected to make the scar scale score. Scar scale score of the study dogs was significantly associated with the day of the image (*p* < 0.0001), and whether laser therapy was used (*p* < 0.001), but not by reviewer (*p* = 0.9). The scar scale rankings were similar for all six veterinary reviewers which consisted of a dermatologist, radiologist, laboratory animal specialist, general practitioner and two surgeons (JG, EB, LS, JB, RB, JW). There were no differences between scar scale scores on days 0, 1, 3, or 5 between groups. There was a statistically significant improvement in scar scores on days 7 and 21 for laser therapy vs. non-laser dogs (*p* < 0.0.1). The mean scar score was significantly higher for the laser (95% CI = 3.21-4.12) than the non-laser (95% CI = 1.85-2.56) dogs at day 7. The mean scar score was significantly higher for the laser (95% CI = 4.52-5.03) than non-laser (95% CI = 3.25-4.21) dogs at day 21 (Figures 2A–C). Dogs on day 21 that received laser therapy had less variation in their score with an average score of 4.78 ± 0.54 vs. non-laser dogs average 3.73 ± 1.34 (Figure 3).

Day 21 results were further compared for laser therapy and non-laser groups for eight patients using the six reviewers. One laser therapy dog did not return for the 21 day photograph. Dogs that received steroid treatment regardless of laser therapy had a clinically significant lower scar score (95% CI 3.41–4.25) vs. dogs that did not receive steroids (mean 5 ± 0.0) at day 21. The dogs that received steroids had a mean scar score that was higher for those that also received laser therapy (95% CI = 4.30-5.32) compared to the steroid administered dogs that were in the non-laser group (95% CI = 2.89-3.94) at day 21. But the two dogs that received steroids and laser therapy had a median score of 5 and a mean score of 4.8 at day 21. Dogs receiving steroids and did not receive laser treatments scored 1–3 points lower on the scar scale at day 21.

**Figure 4:**
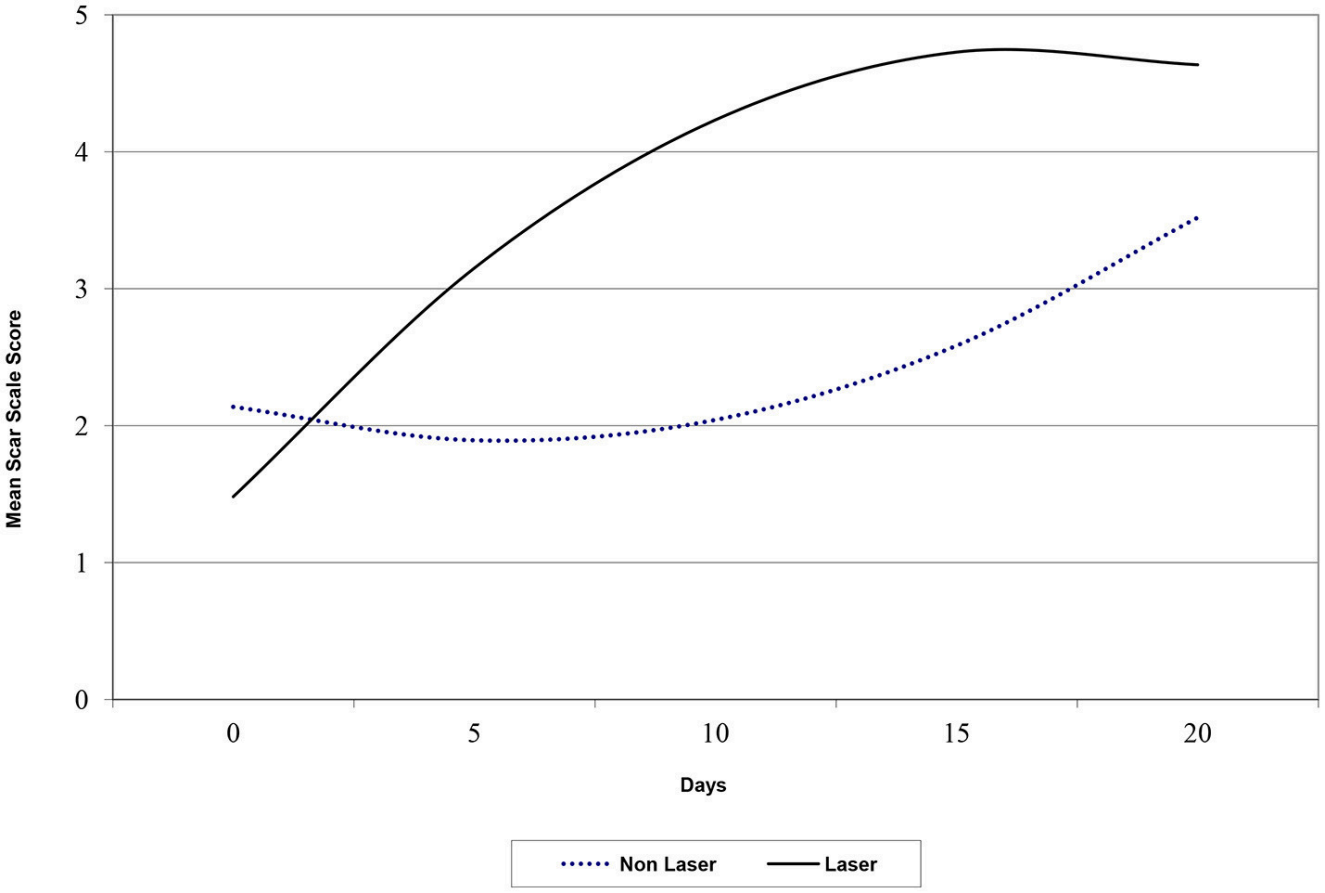
A constant interval graph showing plotted predicted values of scare scale scores for laser treated and non-laser treated patients.

The average score by day 5 was a full point higher on the scar scale score for the laser group and continued to be one point higher on average through day 21. The standard deviation (SD) ranged from 0.85 to 1.6 for all scores except day 21 laser group which had a SD of only 0.35, indicating strong agreement between the reviewers (Table 1). Medians were equally elevated for the laser treated group starting at day 3 and continued until the end of the study. Median scores for the laser group at days 0, 1, 3, 5, 7, and 21 were 1, 2, 2.5, 3, 4, and 5 respectively. The median scar scores for the non-laser group at days 0, 1, 3, 5, 7, and 21 were 1, 2, 2, 2, 2.5, and 4, respectively. Overall there was a strong predictive value for scar scale score with laser therapy treatment in this study (Figure 4). Spearman's Correlation Coefficient was statistically significant for scar score and day for the reviewers (r_s_ = 0.80). Kappa values comparing inter-rater agreement are shown for each day (Table 2).

**Table 1:** The scar scores for the two treatments groups collected on each day.

**Non-Laser**					**Laser**				
**Day**	**Mean**	**SD**	**95% CI**	**Median**	**Day**	**Mean**	**SD**	**95% CI**	**Median**
0	2.12	1.64	1.48–2.76	1	0	1.45	1	1.01–1.89	1
1	2.24	1.17	1.78–2.7	2	1	1.85	1.35	1.26–2.44	2
3	1.92	1.12	1.48–2.36	2	3	2.45	1.1	1.97–2.93	2.5
5	1.72	0.94	1.35–2.09	2	5	3.1	1.29	2.53–3.67	3
7	2.25	0.85	1.88–2.62	2.5	7	3.65	1.14	3.15–4.15	4
21	3.84	1.28	3.34–4.34	4	21	4.87	0.35	4.69–5.04	5

*Mean Confidence intervals (CI) had an alpha set at 0.05. Standard Deviation (sd) for the laser group at day 21 showed very strong agreement among reviewers*.

**Table 2:** Free Marginal Kappa for Inter-rater agreement of scar scores.

	**Kappa coefficient**		
**Day**	**All cases**	**Laser cases**	**Non-laser cases**
0	0.21	0.26	0.17
1	0.21	0.18	0.23
3	0.17	0.14	0.2
5	0.22	0.26	0.18
7	0.16	0.1	0.22
21	0.54	0.79	0.47

## Discussion

Laser therapy has been found to accelerate wound healing by possibly stimulating oxidative phosphorylation therefore reducing the inflammatory response and pain (6, 8–10). This study helps to further support the idea of PBM induced by laser therapy as a vital component to wound healing and rehabilitation by showing the improved outcome of laser therapy on the surgical incisions of dogs with IVDD that underwent a hemilaminectomy.

Five different specialty veterinarians and a general practitioner were recruited and given the same instructions on ranking the overall cosmetic appearance by looking at incision oozing, bruising, crusting, inflammation, edema, granulation, epithelialization, contraction, and hair regrowth on the incision site. Other studies have also evaluated the ability to assess wound healing without using histologic evidence and have found successful modalities, such as a clinical scar scale using digital photography, that are indicated in ante-mortem studies (3, 18, 21). The use of digital photography as a valid means to evaluate the efficacy of wound healing using specific treatment modalities is of particular value because it allows for gross patient evaluation and does not necessitate histologic confirmation (3, 21). This method of evaluation has been more specifically explored using porcine burn scars, human skin grafts and a more recent canine laser study (18, 23, 24). Such studies have confirmed the correlation between histologic characteristics of wound healing with visual assessment of clinical scar outcome. The information extrapolated from this study, and the Gammel et al. (23) study, proves that it is achievable to establish a reliable clinical scar scale using digital photography (23). This high level of correlation therefore indicates that this is a viable method to use when analyzing surgically created wounds treated with ancillary therapy such as PBM induced by laser therapy. We saw a large increase in inter-rater agreement after day 7, especially in the laser group at day 21 having excellent correlation between reviewers (kappa 0.79). While the variance in scar scores improved with healing, the variance in the scores for the laser group was lower throughout the study and had a higher numerical score on the scale throughout the study. This coupled with the excellent kappa value on day 21 demonstrated reference scars are a potentially reliable method to assess clinical healing on photographs. However, higher agreement was seen with increased familiarity with the scar scale score and repeated scoring of photographs (18). Wang et al. demonstrated an increase of correlation coefficients from 65% to over 80% with simply repeated ratings of the photographs. Perhaps, we would have had higher agreement earlier in the results with reviewers scoring the same images several times.

Steroid administration for IVDD patients continues to be a bone of contention among veterinarians. In this prospective study the type, dosage, and length of administration of steroids were not controlled so no conclusions could be drawn. It should also be noted perioperative high dose steroids are not thought to statistically effect wound healing, vs. chronic usage (25). Steroid usage over 10 days in humans can show 2 to 5 times wound complication rates, but varies with comorbidities, dose and surgery (25). We also avoided the laser beam directly over the hemilaminectomy site, due to unwarranted concerns of potential contraindication for laser therapy directly over the spinal cord. This potential concern has been refuted in the literature and has been shown to improve neurologic outcome (4). Combined with our results, these two studies suggest increased surgical healing in IVDD canines using 8 J/cm^2^ daily for the first week after surgery.

Our study used a higher dosage than previously reported for a case report of a chronic canine wound (5 J/cm^2^), for wound healing (5 J/cm^2^), and for open wounds (1 J/cm^2^) (12, 23, 26). It should also be noted that previous studies treated for 4 or 5 days, and this study did for 7 consecutive days (4, 12). An additional study did not have apparent benefits to PBM for therapy 3 times a week for 32 days of treatment using 1 J/cm^2^ (26). This suggests that a higher dose and perhaps a more concentrated treatment schedule may be more successful for PBM. But since these papers were not performed in a parallel manner, it is unclear if our success in speeding wound healing would be seen with an altered protocol.

While every other day laser therapy, more or less J/cm^2^ or less treatment days may alter the findings, canine epidermal keratinocytes showed detrimental effects at 10 J/cm^2^ (27). Since the range in the literature for wound healing is 1–40 J/cm^2^ we chose a middle range, but still an aggressive dosage to increase the chance we would see a benefit, but avoid tissue damage. Recently the World Association of Laser Therapy indicated at least 5–7 J/cm^2^ are needed to induce cellular change, suggesting this to be the low end of therapeutic dosages (28). This is potentially confirmed with the two recent wound healing studies showing no apparent benefit with PBM using 1 or 5 J/cm^2^ in canines (23, 26). In our study, many of the patients were ready to go home prior to the full 7 day treatment was complete. Therefore, the success of laser therapy would need to be weighed against cost to client to remain in hospital or have the patients return for daily outpatient treatments. Non-laser treatment dogs appeared to have a wider scar and more crusting still present, with no hair growth over the incision or bridging epithelium at day 21. Perhaps eventually both groups would look the same further out in time. This is unclear since this study did not follow patients out longer than 21 days.

While this study focused on IVDD patients for a more consistent surgical scar, perhaps this study could be extrapolated to include PBM treatment for other canine surgical incisions or wounds. Similar wounds in the inflammatory phase of wound healing should theoretically respond similarly favorable as in this study. However, since these were uncomplicated, clean, surgical incisions and not open wounds, these conclusions cannot be drawn. It is also unclear if feline patients would need an altered dosage due to their different skin vascularity and healing properties.

A major limitation to this study includes the small sample size, but due to large differences between the groups statistical values were still found which were likely clinically significant. But due to sample size we could not draw conclusion on other interesting variables such as pain, steroid usage, neurologic function, or metabolic variables. This is the first veterinary study to use digital pictures and a scar scale score for surgical incisions. The reviewers were blinded to the treatment groups and pictures were taken uniformly to prevent patient identification by excluding dapple coloring, previous surgery on the back, and cropping into the incision and skin. However, the photographs were colored images and the length of the incisions and axial muscle were not uniform. So, while reviewers were blinded to the treatment group, they may have recognized the shape of an incision or curve of a spine while going through and scoring the randomly numbered images. Therefore, they may have noticed a healing trend on a patient's incision, but they were blinded to the treatment group. The more complete healing, with minimal scarring of wounds, has been shown to coordinate very strongly with overall tensile strength (3, 10, 18, 21, 23, 26). There are obvious incentives to a non-invasive wound healing scoring system. While the true test of tensile strength of wound healing between the two groups would have been more conclusive, the strong agreement between reviewers shows these animals did not need the added morbidity of serial biopsies. This study did not record total time of PBM in each patient. The dosage was predetermined and the incision-encompassing treatment area, but the size of the incision and patient varied; therefore, total joules and treatment times varied. In future studies, this would be a potentially valuable piece of information to record and help uniformity in PBM study reporting and comparison. Additional study limitations were the inability to draw conclusion about many of the things that are known risk factors to abnormal wound healing and operative related factors (i.e., details of scrub, core body temperature, surgery time).

## Conclusion

The surgical incisions in these four dogs healed faster and more cosmetically with PBM induced by laser therapy using 8 J/cm2 daily for 7 days. The improved healing and cosmetic score could be seen beginning at day 7 and continued to be improved for 3 weeks after surgery.

## Authors Contributions

JW, KG, and AW contributed to conception and design of the study. AW organized the database. JW, EB, JB, RB, JG, and LS analyzed the pictures and scored the patients. KG wrote the original grant application for this project. JW wrote the original draft of this manuscript. All authors contributed to manuscript revision, read, and approved the submitted version. The corresponding author takes primary responsibility for communication with the journal and editorial office during the submission process, throughout peer review, and during publication. The corresponding author is also responsible for ensuring that the submission adheres to all journal requirements including, but not exclusive to, details of authorship, study ethics, and ethics approval, clinical trial registration documents and conflict of interest declaration. The corresponding author should also be available post-publication to respond to any queries or critiques.

### Conflict of Interest Statement

The authors declare that the research was conducted in the absence of any commercial or financial relationships that could be construed as a potential conflict of interest.

## Footnotes

^1^Canon PowerShot SD1200 IS Digital ELPH, 10.0 megapixel. Canon U.S.A. Inc. Lake Success, NY 11042, USA.

^2^Vectra Genisys Transport Laser Model 2784. DJO, LLC Vista, CA 92083, USA.

^3^9 Diode Cluster Applicator, 1040 mW total power; (5) 850 nm Laser diodes at 200 mW each, with a spot size of 0.188 cm^2^, and (4) 670 nm LED diodes at 10mW each, with a spot size of 0.64 cm^2^ each. Total contact area 7.55 cm^2^ with a power density of 0.138 W/cm^2^ Hudson Aquatics, Angola IN 46703, USA.

^4^Microsoft Office Excel 2007, Microsoft. Redmond, WA 98052, USA.

^5^SAS Institute. 2008. SAS/STAT Online 9.2 Users Guide. SAS Institute, Cary, NC 27512, USA.

## Abbreviations

PBM: Photobiomodulation
IVDD: Intervertebral Disc Disease.
